# Short sleep and social jetlag are associated with higher intakes of non-milk extrinsic sugars, and social jetlag is associated with lower fibre intakes in those with adequate sleep duration: a cross-sectional analysis from the National Diet and Nutrition Survey Rolling Programme (Years 1–9)

**DOI:** 10.1017/S1368980022000167

**Published:** 2022-09

**Authors:** Haya Al Khatib, Vita Dikariyanto, Kate M Bermingham, Rachel Gibson, Wendy L Hall

**Affiliations:** 1 Department of Nutritional Sciences, School of Life Course and Population Sciences, Faculty of Life Sciences and Medicine, King’s College London, London SE1 9NH, UK; 2 Department of Twin Research and Genetic Epidemiology, School of Life Course and Population Sciences, Faculty of Life Sciences and Medicine, King’s College London, London, UK

**Keywords:** Diet, Sleep, Social jetlag, Adiposity

## Abstract

**Objective:**

To investigate associations and interactions between sleep duration and social jetlag status with nutrient intake, nutrient status, body composition and cardio-metabolic risk factors in a nationally representative UK adult population.

**Design:**

A cross-sectional study using 4-d food diary and self-reported sleep data from the UK National Diet and Nutrition Survey Rolling Programme 2008–2017.

**Setting:**

UK free-living population.

**Subjects:**

Totally, 5015 adults aged 19–64 years.

**Results:**

Thirty-four per cent were short sleepers (< 7 h); 7 % slept ≥ 9 h; 14 % had > 2 h difference in average sleep duration between weeknights and weekend nights (social jetlag). Compared to those reporting optimal sleep duration (≥ 7–< 9 h), short sleep was associated with higher intakes of non-milk extrinsic sugars (NMES) (0·9 % energy, 95 % CI: 0·4, 1·4), total carbohydrate (0·8 % energy, 95 % CI: 0·2, 1·4) and a lower non-starch polysaccharides fibre intake (–0·5 g/d, 95 % CI –0·8, –0·2). There was a significant interaction between short sleep and social jetlag for fibre intakes, where adequate sleepers with social jetlag as well as all short sleepers (regardless of social jetlag) had lower fibre intakes than adequate sleepers with no social jetlag. Short sleep, but not social jetlag, was associated with greater adiposity, but there were no differences in other markers of cardiometabolic disease risk.

**Conclusions:**

The present study reports that both short sleep and social jetlag are associated with higher intakes of NMES, but only sleep duration is associated with markers of adiposity. Social jetlag was associated with lower fibre intakes even in individuals with adequate weekly sleep duration, suggesting catch-up sleep does not prevent the adverse impact of irregular sleep habits on food choices.

Optimal sleep, determined by duration, number of awakenings, consistency with circadian rhythms and presence/absence of clinical sleep disorders, is vital for optimal health and wellbeing^(1)^. Lack of sleep has been highlighted as a public health concern, particularly as current estimates of sleep duration fall short of the recommended 7–9 h per night for adults^(2,3)^. Self-reported short sleep duration (< 7 h per night) has been associated with increased risks of obesity^(4,5)^ and associated cardiometabolic complications^(6–10)^. Long sleep (≥ 9 h) is also associated with relatively poor health outcomes but represents a potentially complex subpopulation where there may be many important confounding factors^(10)^.

As well as average sleep duration, marked variation in intra-individual sleep timing and sleep duration indicates suboptimal sleep habits and misalignment with circadian rhythms and is implicated in risk of poor health^(11,12)^. The term ‘social jetlag’ has been coined^(13)^ to capture a habitual pattern of short sleep during working days relative to longer, catch-up sleep, on free days (usually the weekend). Unlike international travel-related jetlag, social jetlag is a lifelong disrupter of optimal sleep habits and has been positively associated with increased BMI^(14)^ and markers of cardiometabolic disease risk^(15)^.

Proposed mechanisms linking insufficient sleep or circadian disruption with weight gain and metabolic diseases include changes in energy expenditure, hormonal dysregulation and impaired insulin sensitivity^(16,17)^, which may be amplified by modifiable behavioural risk factors^(18)^. As cardiometabolic conditions have strong nutritional determinants, links between poor sleep and metabolic disruption may be mediated through diet^(19)^. Intervention studies under laboratory-controlled conditions have shown that short-term partial sleep deprivation causes higher total energy intakes^(20)^. Chronic exposure to short sleep duration has also been associated with higher energy intakes^(21,22)^, carbohydrate^(23)^, sugar^(24)^ and alcohol intakes^(25)^, as well as lower protein^(24)^, fibre^(21)^ and fruit and vegetable intakes^(26)^, as well as a lower overall diet quality score and unhealthy eating habits^(27–29)^.

Evidence is emerging that dietary patterns and nutrient intakes are also less favourable in those who report social jetlag, including among young university students^(30)^, adolescents^(31)^ and a nationally representative UK adult population (cross-sectional analysis 2008–2011)^(32)^, but it is not clear whether this just reflects chronic sleep deprivation, or whether there is an independent relationship with inconsistency in sleep duration (i.e. social jetlag). The aim of this research is to investigate associations and interactions between sleep duration and social jetlag status with nutrient intake, nutrient status, body composition and cardiometabolic risk factors in a nationally representative UK adult population (2008–2017). The hypothesis was that short sleep duration would be associated with a less healthy dietary profile (higher saturated fat, salt and sugar, lower fibre intakes) and increased adiposity and cardiometabolic risk factors. The second hypothesis was that there would be an interaction between sleep duration and social jetlag.

## Methods

### Study design and cohort

This was a cross-sectional study comparing food intake, nutrient status and cardiometabolic risk factors in short (< 7 h), adequate (≥ 7–< 9 h) and long sleepers (≥ 9 h) in the UK National Diet and Nutrition Survey Rolling Programme (NDNS-RP). The NDNS-RP survey consists of a repeated cross-sectional survey that is carried out every 1·5 years with the aim of assessing nutritional intake and status in a nationally representative UK population^(33)^. The present study investigates the combined results from Years 1–9 of the NDNS (2008/2009–2016/2017) and includes adults aged 19–64 years who are not pregnant or lactating. Full details of the methodology have been previously reported elsewhere^(34)^. In summary, a UK representative random sample of individuals was drawn from a list of all the addresses in the UK, the Postcode Address File. Interviewers administered 4-d food diaries, measured participants’ weight and height, and assisted participants in completing a computer-assisted personal interview (CAPI) to collect background and lifestyle information, including sleep duration.

### Sleep assessment

Sleep duration questions targeted weekdays and weekends separately, phrased as follows: ‘Over the last seven days, that is since (date), how long did you usually sleep for on weeknights. That is Sunday to Thursday nights?’ and ‘Over the last seven days, how long did you usually sleep for on weekend nights. That is Friday and Saturday nights?’ Only the time participants were asleep was included. If participants were on a night shift during the previous 2 weeks, then the average time slept during the day was recorded. Shift work and napping habits were not recorded as part of the NDNS-RP. To estimate a 7-d average sleep duration, the following calculation was applied: ((5 × weeknight sleep duration) + (2 × weekend sleep duration)/7)).

### Dietary assessment and analysis

Participants completed a 4-d estimated food diary with portion sizes were estimated using household measures and weights from labels. To aid accurate estimation, photographs of frequently consumed foods, life-size spoons and a life-size glass were included in the food diary^(34)^. Nutrient intakes were calculated using Diet In Nutrients Out (DINO), a dietary assessment system developed at the Human Nutrition Research Centre at Cambridge University and linked to Public Health England’s NDNS Nutrition Databank^(34)^. Dietary intake outcomes included total energy intake, as well as absolute (g/d) and relative intakes (percentage of total energy intake (%E)) of the macronutrients and alcohol intakes. Dietary fibre was included as defined by the Englyst^(35)^ method, known as non-starch polysaccharides (NSP). As a proxy measure of carbohydrate quality, the carbohydrate:NSP^(36)^ ratio was calculated. Dietary misreporters were identified using McCrory’s method, using 2 sd ^(37)^.

### Nutritional biomarkers

The 24-h urinary excretion of Na, K and N was analysed as objective biomarkers of dietary intake of salt, fruit and vegetables and protein, respectively^(34)^. Circulating nutritional biomarkers included Hb, plasma ferritin, serum vitamin B_12_ and plasma vitamin C, which were measured from blood samples collected during the nurse visit which occurred within 2 to 4 months after dietary data collection.

### Cardiometabolic risk factors

#### Body composition

Participants’ weight and height were measured using a portable weighing scale and stadiometer at the initial interviewer visit. Waist circumference (WC) was measured at the scheduled nurse’s visit upon completion of the 4-d food diary. All measurements were taken twice, and if there were wide variations across measurements (height ± 0·5 cm, weight ± 0·2 kg, WC ± 3 cm), a third was taken and the mean value of the two most similar measurements was reported^(34)^.

#### Blood pressure

Participants were asked to refrain from eating, consuming alcohol or exercising vigorously for 30 min prior to their seated blood pressure measurement. Participants’ blood pressure was measured three times at 1-min intervals between readings using an automated blood pressure monitor (Omron HEM907)^(34)^.

#### Blood measurements

The present study also evaluates cardiometabolic risk using fasting plasma glucose, glycated Hb (HbA1c), serum total cholesterol, HDL-cholesterol, LDL-cholesterol, serum TAG and serum C-reactive protein (CRP)^(34)^.

#### Assessment of confounding variables

Demographics and lifestyle information were collected using the computer-assisted personal interview CAPI^(34)^. This information incorporates age, sex, economic status, qualifications level, the number of children in the household up to 4 years of age, and frequency of alcohol intake and smoking. Information on having any long-standing illness including cancers, mental illness such as anxiety, as well as endocrine, nutritional or metabolic diseases was also collected.

### Statistical analysis

The present study includes adults aged 19–64 years in the NDNS-RP with sleep duration data available. Sleep duration was divided into three categories to define short (< 7 h), adequate (≥ 7–< 9 h) and long (≥ 9 h) sleepers as per National Sleep Foundation recommendations^(38)^.

Previous research using the NDNS data set estimated social jet lag as the difference in sleep duration hours between weekend and weeknights, since midpoint sleep data were not available^(32)^. Participants in this study were categorised as having social jetlag if they reported > 2 h difference in sleep duration between weeknights and weekend nights, based on previous reports showing that this cut-off was associated with increased cardiometabolic risk factors^(14,32,39,40)^.

All statistical analyses were performed using IBM SPSS Statistics 25.0 (Statistical Product and Service Solutions, IBM Corp.). Differences in participant characteristics were assessed using chi-square tests and ANOVA for categorical and scale data, respectively. Normal distributions of residuals were visually checked using histograms and Q-Q plots. Log transformation (ln) was conducted for non-normally distributed residuals. The analysis outcomes were back log-transformed into the geometric mean values. The assumption for homoscedasticity was examined by plotting the standardised residuals of dependent variables and predictors. In sensitivity analyses of outcomes of dietary intake, misreporters were excluded, and the analyses were repeated including only plausible reporters.

The associations between sleep duration or social jetlag category and dietary intake (absolute (g/d) and relative (%EI)), biomarkers of nutrient status, body composition and cardiometabolic risk factors were assessed using ANCOVA, with Bonferroni corrections for post hoc pairwise comparisons. The model was adjusted for age, sex, ethnicity, BMI (except for when assessing BMI and WC as an outcome), total energy intake (except for when assessing energy intake as an outcome), as well as lifestyle and socio-economic confounders including economic status, whether they currently had a long-standing illness, number of children below 4 years of age, smoking status and alcohol intake (except when assessing alcohol as an outcome). A two-sided *P*-value of < 0·05 was considered statistically significant. To account for multiple comparisons, we conducted false discovery rate calculation using the Benjamini and Hochberg method^(41)^. Dietary intake categories (e.g. any marker of dietary carbohydrate quality) and/or cardiometabolic health variables that significantly differed by both sleep duration and social jetlag category were selected, and interactions between sleep duration and social jetlag status were included in the model to reveal independent associations.

Chi-square tests were also conducted to assess the association between food groups and social jetlag. For CRP analysis in investigating the difference between a group with and without social jetlag, multinominal logistics regression was used with adjustments for the same factors as above. CRP values were divided into two groups as follows: < 3 mg/l and ≥ 3–< 10 mg/l.

The NDNS-RP requires survey weighting to correct for differences in sample selection and response. The appropriate weights were used for differential selection probabilities of households and individuals, non-response to the individual questionnaires, non-response to the nurse visit and non-response to the blood sample and urine collection^(34)^. Weight factor wti_Y19 (weight for individual and diary-all ages, combined Years 1–9 (the UK NDNS-RP 2008–2017)) was the weight factor used for examining differences in dietary intakes among short, adequate and long sleeper groups as well as between a group with and without social jetlag; wtn_Y19 (weight for nurse-all ages, combined Years 1–9 (the UK NDNS-RP 2008–2017)) was used for investigating differences in body composition variables (BMI and WC), blood pressure and urinary Na, K and N; and wtb_Y19 (weight for blood-all ages, combined Years 1–9 (the UK NDNS-RP 2008–2017)) was used for investigating differences in blood analyte variables including CRP, lipids, glucose and biomarkers of nutrient status (vitamin B_12_, vitamin C, HbA1C, Hb and serum ferritin).

## Results

### Participant characteristics

Sleep data were available for 5015 adults aged 19–64 years (96% of 5223 total) participating in the NDNS-RP 2008–2017. The characteristics of the cohort in the present study stratified by sleep duration are outlined in Table 1. Short sleepers comprised 34% of the cohort, with only 7% identified as long sleepers. The mean age of the cohort was 42·6 ± 12·4 years, and the mean BMI was 27·5 ± 5·5 kg/m^2^. The cohort was predominantly ‘White’, and 59 % were female. Two-thirds of the cohort were employed (69 %), and 28 % of participants had obtained a degree or equivalent. Approximately two-thirds (67 %) of respondents reported not having any chronic illness, and 82 % had no children between the ages of 0 and 4 years of age living in the household. Current smokers consisted of 26 % of the sample, and 38 % reporting consuming no alcohol. A quarter of respondents (26 %) consumed a dietary supplement.

**Table 1:** Background characteristics of a representative UK adult population (aged 19–64 years) according to sleep duration and social jetlag status based on NDNS 2008–2019 (*n* 5015)

		Sleep duration			*P*-value	FDR value	No social jetlag (*n* 4307)	Social jetlag (*n* 708)	*P*-value	FDR value
Characteristics		< 7 h (*n* 1711)	≥ 7–< 9 h (*n* 2947)	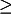 9 h (*n* 357)						
Age										
Mean		45	42	39	< 0·001	< 0·001	43	39	< 0·001	< 0·001
sd		12	12	14			12	12		
Sex, %	Male	42·7	41·4	32·8	0·002	0·003	40·1	48·3	< 0·001	< 0·001
	Female	57·3	58·6	67·2			59·9	51·7		
BMI, kg/m^2^										
Mean		29	27	27	< 0·001	< 0·001	28	27	0·249	0·320
sd		6	5	6			6	5		
Ethnicity, %	White	92·3	90·7	91·8	0·203	0·203	91·3	91·2	0·968	0·968
	Mixed ethnic group	0·9	1·0	1·1			1·0	1·0		
	Black or Black British	2·6	2·3	2·3			2·5	2·1		
	Asian or Asian British	3·5	4·0	3·1			3·7	4·1		
	Any other group	0·7	1·9	1·7			1·5	1·6		
Economic status, %	Going to school or college full time (including on vacation)	2·0	3·8	6·2	< 0·001	< 0·001	3·1	4·8	< 0·001	< 0·001
	In full- or part-time employment	67·0	72·9	44·8			66·9	80·8		
	Not working at present	30·9	23·4	49·0			30·0	14·4		
Children aged between 0 and 4 years, %	0	81·8	81·9	81·5	0·004	0·004	81·2	85·9	0·019	0·029
	1	12·4	14·5	16·0			14·4	11·2		
	2	5·2	3·3	2·2			4·1	2·5		
	3	0·5	0·3	0·3			0·3	0·4		
Smoking status, %	Current cigarette smoker	29·2	23·4	33·2	< 0·001	< 0·001	25·0	33·0	< 0·001	< 0·001
	Ex-regular cigarette smoker	21·3	19·8	17·5			20·6	17·5		
	Never regular cigarette smoker	49·5	56·8	49·3			54·5	49·5		
Alcohol units/d, %	None	38·7	35·5	49·7	< 0·001	< 0·001	38·1	34·6	0·001*	0·002
	≤ 4 (men), 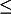 3 (women)	21·8	23·7	17·5			22·7	21·8		
	> 4 and ≤ 8(men), > 3 and 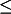 6 (women)	17·5	18·9	11·3			18·2	16·0		
	> 8 (men), > 6 (women)	21·9	21·9	21·5			20·9	27·6		
Long-standing illness present*, %	Yes	37·5	29·0	47·1	< 0·001	< 0·001	33·4	31·8	0·425	0·478
	No	62·5	71·0	52·9			66·6	68·2		
Social jetlag, %		19·3	11·9	7·6	< 0·001	< 0·001				

NDNS, Nutrition Survey Rolling Programme; FDR, false discovery rate.

Data are presented as mean ± sd for continuous variables or *n* (%) for categorical variables. Continuous data were tested by one-way ANOVA. *P* < 0·05 indicates a significant difference between groups. Multiple comparison-adjusted FDR *P*-values using the Benjamini–Hochberg false discovery rate are also provided. Categorical data were tested by chi-square test. *P* < 0·05 indicates a significant association between sleep group and the demographic variables.

* Any physical or mental health condition(s) or illness that have lasted, or expected to last, for 12 months or more.

In the unadjusted stratification of the population by sleep status (Table 1), short sleepers were significantly older and had higher BMI than those who slept ≥ 7 h. A greater proportion of long sleepers were female. There were no differences in the ethnic group distributions between sleep groups. More of the longer sleepers were either going to school or college full-time or not working at present, than the shorter and longer sleepers, with the adequate sleepers having the greatest proportion of people in employment. A greater number of short and long sleepers were either current or ex-smokers. Nearly, half of the long sleepers reported consuming no alcohol, with greater proportions of short and adequate sleepers consuming low or moderate amounts of alcohol. Nearly, half of the long sleepers reported having a long-standing illness, with the lowest proportion of those with long-standing illness being reported in adequate sleepers. Short sleep duration was significantly associated with social jetlag, with 19·3 % of short sleepers being categorised as having social jetlag, compared to only 11·9 % of adequate sleepers and 7·6 % of long sleepers (Table 1).

In the unadjusted stratification of the population by social jetlag status (Table 1), the social jetlag groups were significantly younger, had a greater proportion of men, and were more likely to be current smokers and drink more than 8 units per d (men) or 6 units per d (women) of alcohol than those who had regular sleep duration. BMI and ethnic group distributions did not differ between social jetlag groups. A greater proportion of the social jetlag group were in employment (80·8 %) compared to the no social jetlag group (66·9 %), and there was a slightly larger proportion of the social jetlag group that had no children between the ages of 0 and 4 years old. There were no differences between groups in their reporting of having a long-standing illness.

### Dietary energy, macronutrient and micronutrient intakes

As presented in Table 2, in comparison with adequate sleepers (≥ 7–< 9 h), short sleep (< 7 h) was associated with lower absolute and relative intakes of protein and *n*-3 PUFA. There were no significant differences in intakes of saturated or trans fats in short sleepers in comparison with adequate sleepers. Short sleep was also associated with higher absolute and relative intake of carbohydrates, non-milk extrinsic sugars (NMES), and a higher carbohydrate:NSP ratio. In addition, short sleep was associated with lower intake of NSP fibre, folate, vitamin B_12_, vitamin C, K, Fe, and fruits and vegetables in comparison with adequate sleep. However, biomarkers of intake for vitamin B_12_, vitamin C, Fe (Hb and serum ferritin), and urinary Na (salt), K and N (protein) excretion were not significantly different between short sleepers and adequate sleepers (online supplementary material, Supplemental Table 1). In comparison with adequate sleepers (≥ 7–< 9 h), long sleepers (≥ 9 h) had no differences in dietary intakes (Table 2).

**Table 2:** Daily energy, macro- and micronutrient intake (estimated marginal mean (95 % CI)) of short, adequate and long sleepers*, in the UK adult population (aged 19–64 years) based on NDNS 2008–2019

	Short sleep, < 7 h				Adequate sleep, 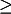 7–< 9 h		Long sleep, 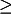 9 h			
	Estimated marginal mean	95 % CI	*P*-value (short *v*. adequate sleep)	FDR value	Estimated marginal mean	95 % CI	Estimated marginal mean	95 % CI	*P*-value (long *v*. adequate sleep)	FDR value
Total energy (kcal)	1744	1657, 1831	0·026‡	0·068	1791	1704, 1878	1684	1579, 1788	0·004‡	0·108
Total energy (kJ)	7337	6972, 7703	0·027‡	0·068	7534	7169, 7900	7084	6647, 7521	0·004‡	0·108
Protein (g)†	68·9	66·4, 71·4	< 0·001‡	< 0·001§	71·6	69·1, 74·2	70·6	67·6, 73·7	0·929	1·000
Protein (%EI)†	16·5	15·9, 17·0	< 0·001‡	< 0·001§	17·0	16·4, 17·6	16·5	16·0, 17·4	0·552	1·000
Fat (g)	70·0	68·0, 71·9	0·723	1·000	70·4	68·5, 72·4	69·4	67·1, 71·8	0·525	1·000
Fat (%EI)	34·4	33·4, 35·4	0·369	0·710	34·7	33·7, 35·7	34·1	32·9, 35·3	0·405	1·000
Monounsaturated fat (g)	25·9	25·0, 26·8	1·000	1·000	26·0	25·1, 27·0	25·9	24·8, 27·0	1·000	1·000
Monounsaturated fat (%EI)	12·7	12·2, 13·2	0·788	1·000	12·8	12·3, 13·2	12·6	12·1, 13·2	1·000	1·000
*n*-3 fatty acids (g)†	1·9	1·8, 2·0	0·001‡	0·004§	2·0	1·8, 2·1	1·9	1·7, 2·0	0·196	1·000
*n*-3 fatty acids (%EI)	1·0	1·0, 1·1	0·008‡	0·024§	1·1	1·0, 1·1	1·0	0·9, 1·1	0·219	1·000
*n*-6 fatty acids (g)	10·5	10·0, 11·0	0·368	0·710	10·7	10·2, 11·2	10·7	10·1, 11·3	1·000	1·000
*n*-6 fatty acids (%EI)	5·2	5·0, 5·5	0·055	0·125	5·4	5·1, 5·6	5·3	5·0, 5·6	1·000	1·000
Saturated fat (g)	25·0	24·0, 26·1	1·000	1·000	25·0	24·0, 26·1	24·4	23·1, 25·6	0·296	1·000
Saturated fat (%EI)	12·2	11·7, 12·7	1·000	1·000	12·2	11·7, 12·7	11·9	11·3, 12·6	0·446	1·000
Trans fat (g)†	1·0	0·9, 1·1	0·477	0·883	1·0	0·9, 1·1	1·0	0·9, 1·1	0·071	0·888
Trans fat (%EI)†	0·5	0·5, 0·6	0·830	1·000	0·5	0·5, 0·6	0·5	0·5, 0·6	0·183	1·000
Carbohydrate (g)	227·5	222·1, 233·0	0·017‡	0·047§	224·4	219·0, 229·8	227·9	221·3, 234·4	0·288	1·000
Carbohydrate (%EI)	48·9	47·8, 50·1	0·002‡	0·008§	48·1	47·0, 49·3	49·0	47·7, 50·4	0·116	1·000
NMES (g)	57·0	52·0, 61·9	< 0·001‡	< 0·001§	52·3	47·3, 57·3	53·8	47·8, 59·7	1·000	1·000
NMES (%EI)	11·9	10·8, 12·9	< 0·001‡	< 0·001§	11·0	9·9, 12·0	11·3	10·1, 12·6	1·000	1·000
Alcohol (g)	10·2	7·0, 12·4	0·743	1·000	9·4	6·2, 12·6	8·5	4·7, 12·4	1·000	1·000
Alcohol (%EI)	3·1	2·1, 4·2	1·000	1·000	3·0	2·0, 4·0	2·7	1·5, 3·9	1·000	1·000
NSP (g)†	12·6	12·0, 13·3	< 0·001‡	0·002§	13·1	12·5, 13·8	13·2	12·4, 13·9	1·000	1·000
Carbohydrate:NSP†	17·2	16·3, 18·0	< 0·001‡	< 0·001§	16·4	15·6, 17·3	16·8	15·8, 17·8	0·815	1·000
Folate (μg)†	220·1	208·5, 232·3	0·001‡	0·004§	229·3	217·0, 242·0	237·7	222·7, 253·7	0·243	1·000
Vitamin B_12_ (μg)†	4·3	4·0, 4·7	0·002‡	0·008§	4·6	4·2, 4·9	4·4	4·0, 4·9	1·000	1·000
Vitamin C (mg)†	64·1	57·6, 71·2	< 0·001‡	< 0·001§	71·2	63·9, 79·1	71·6	63·1, 81·3	1·000	1·000
K (mg)†	2514·9	2426·0, 2673·1	0·002‡	0·008§	2578·6	2487·4, 2673·1	2581·2	2470·1, 2694·6	1·000	1·000
Ca (mg)†	678·6	648·1, 709·8	0·722	1·000	671·2	641·0, 702·0	679·3	643·6, 716·9	1·000	1·000
Fe (mg)†	9·7	9·3, 10·1	< 0·001‡	< 0·001§	10·1	9·7, 10·5	10·2	9·7, 10·8	1·000	1·000
Fruits and Vegetables (g)†	239·3	214·3, 267·0	< 0·001‡	< 0·001§	268·6	240·8, 299·7	239·3	209·8, 272·7	0·017‡	0·288

NDNS, Nutrition Survey Rolling Programme; FDR, false discovery rate; NMES, non-milk extrinsic sugars; %EI, % energy intake; NSP, non-starch polysaccharides.

ANCOVA was used adjusted for age, sex, BMI, total energy intake (except for when assessing energy intake as the outcome), ethnicity, economic status, smoking status, alcohol intake (except when assessing alcohol as an outcome), number of children below 4 years of age and long-standing illness.

* Short, adequate and long sleepers *n* 1431, 2527 and 297, respectively.

† Residual data are non-normally distributed. The values presented from these variables are geometric (back log-transformed) means with 95 % CI.

‡ Two-sided *P* < 0·05 shows a significant difference.

§ Significant difference using multiple comparison-adjusted FDR *P*-values using the Benjamini–Hochberg false discovery rate.

Results of a sensitivity analysis in plausible dietary intake reporters only (85·2 % of the cohort, *n* 4274), as presented in Supplemental Table 2, show similar results to the analysis of the whole cohort, including significantly greater NMES intakes and a higher carbohydrate:NSP ratio in short sleepers compared with adequate sleepers. However, unlike the whole sample, both short and long sleepers reported lower energy intakes than adequate sleepers, and there were no differences in total carbohydrate intake (absolute or relative) in plausible reporters. In addition, there was no association between short sleep and K intake in plausible reporters.

Although short sleep was associated with higher NMES intake, the proportion of consumers of high sugar foods (‘sugar confectionery’, ‘sugars, preserves and sweet spreads’, ‘soft drinks, not low calorie’, ‘puddings’) was similar between the short sleep and adequate sleep groups (online supplementary material, Supplemental Table 3), or higher in the adequate sleep group (‘buns, cakes, pastries and fruit pies’, ‘chocolate confectionery’). However, a greater proportion of adequate sleepers consumed ‘brown, granary and wheatgerm bread’, ‘cheese’, ‘eggs and egg dishes’, ‘fruit’, ‘fruit juice’, ‘nuts and seeds’, ‘oily fish’, ‘other white fish, shellfish and fish dishes’, ‘salad and other raw vegetables’, ‘savoury sauces, pickles, gravies, and condiments’, ‘semi-skimmed milk’, ‘vegetables, not raw’ and ‘wine’ compared to short and long sleep groups.

As presented in Table 3, there were no significant differences in intakes of energy, protein, fat, total carbohydrate or alcohol between those with social jetlag compared with no social jetlag. However, social jetlag was associated with higher absolute NMES intakes and lower relative intakes of SFA, and absolute and relative intakes of trans fatty acids. There were no differences in NSP fibre or carbohydrate:NSP ratio, nor any differences in vitamin and mineral intakes, fruits and vegetables intake (Table 3), nor biomarkers of salt, K, protein, Fe, vitamin C and vitamin B_12_ intake (online supplementary material, Supplemental Table 4).

**Table 3:** Daily energy, macro- and micronutrient intake (estimated marginal mean (95 % CI)) of a group with and without social jetlag*, in the UK adult population (aged 19–64 years) based on NDNS 2008–2019

	No social jetlag		Social jetlag			
Dietary intake	Estimated marginal mean	95 % CI	Estimated marginal mean	95 % CI	*P*-value	FDR value
Total energy (kcal)	1762	1676, 1848	1757	1663, 1851	0·832	0·961
Total energy (kJ)	7412	7052, 7771	7393	6998, 7788	0·853	0·961
Protein (g)†	70·2	67·8, 72·8	70·0	67·3, 72·8	0·733	0·961
Protein (%EI)†	16·7	16·2, 17·3	16·7	16·1, 17·4	0·901	0·961
Fat (g)	70·3	68·4, 72·2	69·4	67·2, 71·5	0·082	0·241
Fat (%EI)	34·6	33·6, 35·6	33·9	32·8, 35·0	0·011‡	0·081
Monounsaturated fat (g)	26·0	25·1, 26·9	26·0	25·0, 27·0	0·866	0·961
Monounsaturated fat (%EI)	12·7	12·3, 13·2	12·6	12·1, 13·1	0·553	0·961
*n*-3 fatty acids (g)†	1·93	1·81, 2·05	1·86	1·73, 1·99	0·039‡	0·178
*n*-3 fatty acids (%EI)	1·1	1·0, 1·1	1·0	0·9, 1·1	0·054	0·193
*n*-6 fatty acids (g)	10·6	10·1, 11·1	10·5	10·0, 11·1	0·725	0·961
*n*-6 fatty acids (%EI)	5·3	5·1, 5·6	5·3	5·0, 5·6	0·637	0·961
Saturated fat (g)	25·1	24·1, 26·1	24·5	23·4, 25·6	0·035‡	0·177
Saturated fat (%EI)	12·3	11·8, 12·8	11·9	11·3, 12·4	0·003‡	0·041§
Trans fat (g)†	1·04	0·96, 1·12	0·96	0·88, 1·04	< 0·001‡	0·002§
Trans fat (%EI)†	0·53	0·49, 0·57	0·49	0·45, 0·53	< 0·001‡	0·002§
Carbohydrate (g)	225·7	220·3, 231·0	228·7	222·8, 234·5	0·044‡	0·178
Carbohydrate (%EI)	48·5	47·4, 49·6	49·1	47·9, 50·4	0·033‡	0·177
NMES (g)	54·0	49·1, 58·9	58·3	53·0, 63·7	0·001‡	0·022§
NMES (%EI)	11·3	10·3, 12·3	12·1	11·0, 13·2	0·006‡	0·063
Alcohol (g)	10·0	6·9, 13·2	8·0	4·6, 11·5	0·023‡	0·146
Alcohol (%EI)	3·1	2·1, 4·1	2·6	1·5, 3·7	0·071	0·237
NSP fibre (g)†	12·9	12·3, 13·5	12·7	12·1, 13·4	0·350	0·729
Carbohydrate:NSP (g)†	16·7	15·9, 17·6	17·1	16·2, 18·1	0·080	0·241
Folate (μg)†	225·9	214·0, 238·2	222·5	209·8, 235·8	0·311	0·707
Vitamin B_12_ (μg)†	4·4	4·1, 4·8	4·4	4·0, 4·8	0·697	0·961
Vitamin C (mg)†	67·7	60·9, 75·2	67·6	60·3, 75·9	0·966	0·978
K (mg)†	2547·8	2460·2, 2641·2	2540·2	2443·0, 2641·2	0·700	0·961
Ca (mg)†	675·2	646·1, 706·3	673·8	641·6, 707·7	0·891	0·961
Fe (mg)†	9·9	9·5, 10·3	9·7	9·3, 10·2	0·115	0·303
Fruit and vegetables (g)†	253·2	227·1, 281·9	246·9	219·1, 277·9	0·394	0·788

NDNS, Nutrition Survey Rolling Programme; FDR, false discovery rate; %EI, % energy intake; NMES, non-milk extrinsic sugars; NSP, non-starch polysaccharides.

ANCOVA was used adjusted for age, sex, BMI, total energy intake (except for when assessing energy intake as the outcome), ethnicity, economic status, smoking status, alcohol intake (except when assessing alcohol as an outcome), number of children below 4 years of age and long-standing illness.

* Without social jetlag *n* 3638; with social jetlag *n* 617.

† Residual data are non-normally distributed. The values presented from these variables are geometric (back log-transformed) means with 95 % CI.

‡ Two-sided *P* < 0·05 shows a significant difference.

§ Significant difference using multiple comparison-adjusted FDR *P*-values using the Benjamini–Hochberg false discovery rate.

A greater proportion of the social jetlag group reported consuming ‘chocolate confectionery’, ‘crisps and savoury snacks’ and ‘other meat and meat products’ (online supplementary material, Supplemental Table 5). A smaller proportion of the social jetlag group consumed ‘fruit’, ‘oily fish’, ‘other potatoes, potato salads, dishes’ (i.e. not including potato chips, fried roast potatoes and potato products’), and ‘tea, coffee and water’ compared to those not reporting social jetlag.

Short sleep was associated with a higher BMI and WC in comparison with the adequate sleep group (Table 4), but there were no differences between groups for blood pressure, fasting glucose concentrations, HbA1c, blood lipids or CRP. There were no differences in any of the cardiometabolic risk factors between the long sleep and adequate sleep groups. Body composition was not associated with social jetlag status, and there were no other differences in markers of cardiometabolic risk in the social jetlag group compared with the no social jetlag group.

**Table 4:** Cardiometabolic risk (estimated marginal mean (95 % CI)) in a representative UK adult population (aged 19–64 years)* according to sleep duration and social jetlag status based on NDNS 2008–2019

	Short sleep, < 7 h				Adequate sleep, 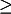 7–< 9 h		Long sleep, 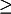 9 h				No social jetlag		Social jetlag			
Cardiometabolic risk factor	Estimated marginal mean	95 % CI	*P*-value (short *v*. adequate sleep)	FDR value	Estimated marginal mean	95 % CI	Estimated marginal mean	95 % CI	*P*-value (long *v*. adequate sleep)	FDR value	Estimated marginal mean	95 % CI	Estimated marginal mean	95 % CI	*P*-value	FDR value
BMI (kg/m^2^)	28·4	27·4, 29·4	< 0·001‡	< 0·001||	27·1	26·2, 28·1	26·8	25·6, 27·9	0·982	1·000	27·7	26·7, 28·6	27·8	26·7, 28·8	0·602	0·961
WC (cm)	93·9	90·3, 97·5	0·005‡	0·017||	91·6	88·0, 95·2	89·5	85·2, 93·9	0·401	1·000	94·3	92·0, 96·7	95·1	92·6, 97·6	0·231	0·550
SBP (mmHg)	120·4	117·8, 123·0	0·062	0·135	121·8	119·2, 124·4	123·5	120·3, 126·7	0·361	1·000	121·3	118·8, 123·9	121·2	118·3, 124·0	0·851	0·961
DBP (mmHg)	73·3	71·3, 75·3	1·000	1·000	73·5	71·5, 75·5	74·8	72·4, 77·3	0·325	1·000	73·5	71·5, 75·4	73·6	71·5, 75·8	0·786	0·961
Glucose (mmol/l)	5·2	4·9, 5·5	0·649	1·000	5·2	4·9, 5·5	5·2	4·8, 5·5	1·000	1·000	5·2	5·0, 5·4	5·2	4·9, 5·4	0·895	0·961
HbA1C (%)†	5·6	5·4, 5·7	0·038‡	0·091	5·5	5·4, 5·7	5·6	5·4, 5·8	0·145	1·000	5·5	5·4, 5·7	5·5	5·4, 5·7	0·926	0·965
TC (mmol/l)	5·0	4·7, 5·3	1·000	1·000	5·0	4·7, 5·3	4·9	4·6, 5·3	1·000	1·000	5·0	4·7, 5·3	4·9	4·6, 5·2	0·046‡	0·178
TAG (mmol/l)†	1·1	1·0, 1·3	1·000	1·000	1·1	1·0, 1·3	1·1	1·0, 1·3	1·000	1·000	1·1	1·0, 1·3	1·1	1·0, 1·3	0·903	0·961
HDL (mmol/l)	1·5	1·4, 1·6	1·000	1·000	1·5	1·3, 1·6	1·5	1·3, 1·6	1·000	1·000	1·5	1·4, 1·6	1·4	1·3, 1·5	0·009‡	0·078
LDL (mmol/l)	3·0	2·7, 3·2	1·000	1·000	3·0	2·7, 3·2	2·9	2·7, 3·2	1·000	1·000	2·9	2·6, 3·1	2·8	2·6, 3·1	0·443	0·852
TC:HDL†	3·4	3·2, 3·7	1·000	1·000	3·4	3·1, 3·7	3·4	3·1, 3·7	1·000	1·000	3·4	3·2, 3·7	3·5	3·2, 3·8	0·325	0·707
CRP (mg/l)†,§	1·7	1·4, 2·1	1·000	1·000	1·7	1·4, 2·1	1·7	1·5, 2·3	1·000	1·000	1·7	1·4, 2·1	1·6	1·3, 1·9	0·978	0·978

NDNS, Nutrition Survey Rolling Programme; FDR, false discovery rate; WC, waist circumference; SBP, systolic blood pressure; DBP, diastolic blood pressure; HbA1C, glycated Hb; TC, total cholesterol; CRP, C-reactive protein.

* BMI: short, adequate and long sleepers *n* 1082, 1930 and 215, respectively; without social jetlag *n* 2755; *n* with social jetlag *n* 472. WC: short, adequate, and long sleepers *n* 869, 1594 and 178, respectively; without social jetlag *n* 2824; with social jetlag *n* 484. SBP and DBP: short, adequate and long sleepers *n* 847, 1534 and 164, respectively; without social jetlag *n* 2181; with social jetlag *n* 364. ANCOVA was used adjusted for age, sex, BMI, ethnicity, economic status, smoking status, alcohol intake, number of children below 4 years of age and long-standing illness.

Glucose: short, adequate and long sleepers *n* 708, 1279 and 125, respectively; without social jetlag *n* 1821; with social jetlag *n* 291. HbA1C: short, adequate and long sleepers *n* 715, 1294 and 134, respectively; without social jetlag *n* 1835; with social jetlag *n* 308. TC, HDL and TC:HDL: short, adequate and long sleepers *n* 737, 1350 and 134, respectively; without social jetlag *n* 1908; with social jetlag *n* 313. TAG: short, adequate and long sleepers *n* 735, 1348 and 134, respectively; without social jetlag *n* 1905; with social jetlag *n* 312. LDL: short, adequate and long sleepers *n* 728, 1326 and 134, respectively; without social jetlag *n* 1882; with social jetlag *n* 306. CRP: short, adequate and long sleepers *n* 738, 1350 and 134, respectively; without social jetlag *n* 1909; with social jetlag *n* 313.

† Residual data are non-normally distributed. The values presented from these variables are geometric (back log-transformed) means with 95 % CI.

‡ Two-sided *P* < 0·05 shows a significant difference.

§ Multinominal logistics regression was used adjusted for age, sex, BMI, ethnicity, economic status, smoking status, alcohol intake, number of children below 4 years of age and long-standing illness. CRP values were divided into two groups: < 3 mg/l and ≥ 3–< 10 mg/l.

|| Significant difference using multiple comparison-adjusted FDR *P*-values using the Benjamini–Hochberg false discovery rate.

### Sleep duration and social jetlag interactions

Since NMES (g) intakes were higher in the short sleep and social jetlag groups, and NSP intakes were lower in short sleepers only, interactions between sleep duration and social jetlag were investigated for indicators of carbohydrate quality. There was a significant sleep duration × social jetlag interaction for NSP intake (*P* = 0·022). Geometric means (95 % CI) for NSP fibre in adequate sleepers were significantly higher in those with no social jetlag (13·2 g/d (12·5, 13·8), *n* 2596) compared those with social jetlag (12·7 g/d (12·0, 13·5), *n* 351, *P* = 0·043), whereas fibre intake was lower overall in short sleepers compared with adequate sleepers with no social jetlag but did not differ within the short sleep group according to social jetlag category (no social jetlag 12·6 g/d (12·0, 13·2), *n* 1381; social jetlag 12·9 g/d (12·2, 13·6), *n* 330, *P* = 0·221). There were no significant interactions between sleep duration and social jetlag for total carbohydrate, NMES or carbohydrate:NSP ratio.

## Discussion

This cross-sectional study examined whether both sleep duration and social jetlag were associated with dietary intakes and cardiometabolic risk factors in a representative sample of UK adults, and further investigated whether there were independent effects of social jetlag regardless of sleep duration. Consistent with previous evidence^(28,42)^, we report that approximately one-third of the UK adult population are not achieving recommended sleep durations, and 14 % were experiencing social jetlag, as defined by a difference in weeknight and weekend night sleep duration < 2 h. As expected, we found a higher prevalence of social jetlag in short sleepers in comparison with those achieving adequate sleep^(32)^.

Nutrient intakes in short sleepers suggested a less healthy diet, characterised by higher intake of added sugars and lower intake of NSP fibre, *n*-3 fatty acids, protein, fruits and vegetables, and specific micronutrients, compared with adequate sleepers. A previous analysis of the NDNS-RP years 1–4 reporting on linear associations of dietary intake and sleep duration without adjustment for total energy intake found no significant associations between sleep and nutrient intakes^(43)^, which might be explained by the relationship between sleep and diet being non-linear^(19)^. Whilst habitual sleep deficiency may promote hedonic cravings for sweet, energy-dense foods resulting in poorer health outcomes, populations with average sleep durations > 9 h might sleep longer because of long-standing health conditions, thus confounding associations with diet.

Short sleep was associated with higher carbohydrate intakes and poorer quality carbohydrate intake, evidenced by higher absolute and relative intake of NMES and a higher carbohydrate:NSP ratio, which may lead to greater risk of weight gain. Indeed, short sleep was associated with higher adiposity measures, in agreement with a meta-analysis of twelve prospective cohort studies that followed up from 1 to 12 years showing that short sleep duration was associated with greater risk of obesity^(44)^. Short sleepers may be less physically active^(45)^ leading to a net positive energy balance. However, it could have also been mediated by higher consumption of energy-dense, fibre-poor foods. Sleep-deprived subjects had alterations in neuronal responses in areas of the brain linked to reward, which would be expected to result in a heightened preference for energy-dense, palatable foods^(46,47)^, alongside increased ghrelin secretion^(47,48)^. Therefore, extending sleep duration could reduce cravings for energy-dense, low-fibre foods, and in fact we observed a decreased intake in free sugars following a sleep extension intervention in short sleepers^(49)^. Alongside previous observational evidence^(21,23,24)^, it appears likely that higher dietary sugars and low dietary fibre are the key aspects of diet quality that may predispose to weight gain when sleep duration is habitually inadequate.

Social jetlag was also associated with higher sugar intakes. However, the social jetlag group differed from short sleepers in that they also had lower relative mean intakes of saturated and trans fatty acids and had none of the other significant differences in dietary intake nor cardiometabolic risk factors. A significant interaction between sleep duration and social jetlag revealed that NSP fibre intake was not modified by social jetlag status in short sleepers, but that intakes were lower in adequate sleepers with social jetlag than those without social jetlag. This suggests that both short sleep and sleep inconsistency between week and weekend nights may influence dietary carbohydrate quality independently, although it is also possible that reverse causation was at work and lower fibre intakes might somehow reduce sleep duration.

There are some limitations in this cross-sectional study. Temporal trends cannot be ascertained and no causal inferences or directions of effect can be drawn. Measures of sleep duration and dietary intake rely on self-report, which may be inaccurate. The difference in sleep duration between weeknights and weekend nights was used as a proxy measure for social jet lag, a method applied in observational studies when sleep-onset and wake-up data are not available to calculate difference in sleep midpoint between ‘work nights’ and ‘free nights’^(32)^. Social jetlag was calculated from the self-reported difference in sleep durations between the typical working weeknights and weekend nights, but not all participants would have worked Monday to Friday with weekends off^(13)^. No information was available on shift work status and duration or hours of work; these may implicate circadian disruption via different mechanisms. In individuals not currently in employment, retirement status was not considered, nor menopausal status in women. Finally, later time of eating and a longer eating window have been associated with increased cardiometabolic risk and may impact on sleep duration^(50)^. The present study did not investigate these additional dietary variables but should be a consideration for future research.

## Conclusion

In conclusion, the present study reports that short sleep and social jetlag are associated with higher intake of sugar in a representative UK population sample. Furthermore, the association between short sleep and low fibre intake is extended to those with social jetlag even if they have adequate average weekly sleep duration, suggesting that catch-up sleep does not mitigate the effects of irregular sleep durations on diet quality. This study corroborates previous findings that short sleepers exhibit higher markers of adiposity but found no independent effects of social jetlag on body composition in the adjusted analysis. Combined with increasingly sedentary behaviours of the modern lifestyles, poor dietary carbohydrate quality in short sleepers and those with social jetlag may increase the risk of weight gain and cardiometabolic diseases over time. Prospective cohort studies utilising nutritional biomarkers of carbohydrate quality and objective measures of sleep are needed to address the potential lifelong impact of modest circadian misalignments associated with modern working lifestyles, which affect a large proportion of the global population.

## Supplementary material

For supplementary material accompanying this paper visit https://doi.org/10.1017/S1368980022000167. click here to view supplementary material
