# Supplementary material for: Short sleep and social jetlag are associated with higher intakes of non-milk extrinsic sugars, and social jetlag is associated with lower fibre intakes in those with adequate sleep duration: a cross-sectional analysis from the National Diet and Nutrition Survey Rolling Programme (Years 1–9)
Source: Public Health Nutr. 2022 Jan 18;25(9):2570–81. doi: 10.1017/S1368980022000167 (PMC9991673; doi:10.1017/S1368980022000167)
Supplement: Supplementary file 1 [file S1368980022000167sup001.docx]

Supplementary Table 1. Biomarkers of nutrient intakes (estimated marginal mean (95% CI)) of short, adequate and long sleepers in the UK adult population (19-64 y) based on NDNS 2008–2019 (n=5,015).

|  | **Short sleep, <7 h** | ***P* value (short vs adequate sleep)** | **FDR value** | **Adequate sleep,** $\boldsymbol{\geq}$**7 - <9 h** | **Long sleep,** $\boldsymbol{\geq}$**9h** | ***P* value (long vs adequate sleep)** | **FDR value** |
| --- | --- | --- | --- | --- | --- | --- | --- |
| Vitamin B_12_ (pmol/l)^1^ | 266.4 (242.7, 292.7) | 1.000 | 1.000 | 262.7 (239.4, 288.3) | 269.3 (241.5, 300.4) | 1.000 | 1.000 |
| Vitamin C (μmol/l) | 64.3 (51.0, 77.6) | 0.562 | 0.969 | 61.9 (48.7, 75.0) | 58.9 (48.7, 74.3) | 1.000 | 1.000 |
| Haemoglobin (g/dl) | 13.8 (13.5, 14.1) | 1.000 | 1.000 | 13.8 (13.6, 14.1) | 13.7 (13.4, 14.0) | 0.926 | 1.000 |
| Serum ferritin (μg/l)^1^ | 63.8 (46.4, 87.7) | 0.226 | 0.471 | 68.2 (49.7, 93.7) | 66.4 (46.9, 93.9) | 1.000 | 1.000 |
| Urinary Na^+1^ excretion (mmol/24hrs) | 114.5 (97.9, 131.0) | 1.000 | 1.000 | 117.1 (100.5, 133.6) | 111.3 (91.2, 131.5) | 1.000 | 1.000 |
| Urinary K^+^ excretion (mmol/24hrs)^1^ | 58.9 (52.6, 65.9) | 1.000 | 1.000 | 59.9 (53.6, 67.1) | 59.4 (51.8, 68.1) | 1.000 | 1.000 |
| Urinary N^3-^ excretion (g/24hrs)^2^ | 10.6 (9.7, 11.7) | 0.498 | 0.889 | 10.9 (10.0, 12.0) | 10.3 (9.2, 11.5) | 0.227 | 1.000 |

Vitamin B_12_ (pmol/l): short-, adequate- and long-sleepers n=740, 1314, 129 respectively; Vitamin C (μmol/l): short-, adequate- and long-sleepers n=276, 492, 42; Haemogloblin: short-, adequate- and long-sleepers n=725, 1750, 194; Serum ferritin: short-, adequate- and long-sleepers n=737, 1319, 133; Urinary K^+^, N^3^ and Na^+1^: short-, adequate- and long-sleepers n=376, 714, 75.

ANCOVA was used adjusted for age, sex, body mass index (BMI), ethnicity, economic status, smoking status, alcohol intake, number of children below 4 years, and long standing illness.

^1^Residual data are non-normally distributed. The values presented from these variables are geometric (back log transformed) means with 95% CI.

Supplementary Table 2. Daily energy, macro- and micronutrient intake (estimated marginal mean (95% CI)) of short, adequate, and long sleepers who were plausible reporters, in the UK adult population (19-64 y) based on NDNS 2008–2019 (n = 4,274).

| **Dietary intake** | **All (n_max_ = 4,274)** | **Short sleep, <7 h (n = 1,430)** | ***P* value** | **FDR value** | **Adequate sleep,** $\boldsymbol{\geq}$**7 - <9 h (n = 2,537)** | **Long sleep,** $\boldsymbol{\geq}$ **9 h (n = 307)** | ***P* value** | **FDR value** |
| --- | --- | --- | --- | --- | --- | --- | --- | --- |
| Total energy (kcal) | 1784 ± 568 | 1768.2 (1675.7, 1860.7) | 0.022^2^ | 0.049^3^ | 1818.3 (1725.6, 1911.0) | 1703.3 (1594.8, 1811.7) | 0.002^2^ | 0.034^3^ |
| Total energy (kJ) | 7500 ± 2381 | 7437.6 (7049.5, 7825.6) | 0.022^2^ | 0.049^3^ | 7646.9 (7258.1, 8035.7) | 7166.2 (6711.3, 7621.1) | 0.002^2^ | 0.034^3^ |
| Protein (g)^1^ | 70.0 (29.6) | 68.7 (66.1, 71.5) | <0.001^2^ | 0.002^3^ | 71.0 (68.2, 73.8) | 70.4 (67.2, 73.7) | 1.000 | 1.000 |
| Protein (%EI)^1^ | 16.9 (4.8) | 16.7 (16.1, 17.3) | 0.006^2^ | 0.021^3^ | 17.1 (16.5, 17.7) | 16.9 (16.2, 17.6) | 1.000 | 1.000 |
| Fat (g) | 64.3 (34.3) | 69.8 (67.7, 71.9) | 1.000 | 1.000 | 70.0 (67.9, 72.1) | 69.1 (66.6, 71.5) | 0.713 | 1.000 |
| Fat (%EI) | 34.8 ± 6.5 | 34.9 (33.8, 36.0) | 1.000 | 1.000 | 35.0 (33.9, 36.1) | 34.5 (33.2, 35.8) | 0.504 | 1.000 |
| Monounsaturated fat (g) | 23.3 (12.9) | 25.8 (24.8, 26.8) | 1.000 | 1.000 | 25.8 (24.8, 26.8) | 25.7 (24.5, 26.8) | 1.000 | 1.000 |
| Monounsaturated fat (%EI) | 12.8 ± 4.5 | 12.8 (12.3, 13.4) | 1.000 | 1.000 | 12.9 (12.4, 13.4) | 12.8 (12.2, 13.4) | 1.000 | 1.000 |
| n-3 fatty acids (g) | 1.7 (1.2) | 2.1 (2.0, 2.3) | 0.039^2^ | 0.081 | 2.2 (2.1, 2.4) | 2.1 (1.9, 2.3) | 0.162 | 0.628 |
| n-3 fatty acids (%EI)^1^ | 1.0 (0.5) | 1.0 (0.9, 1.0) | 0.009^2^ | 0.029^3^ | 1.0 (0.9, 1.1) | 1.0 (0.9, 1.0) | 0.157 | 0.628 |
| n-6 fatty acids (g) | 8.9 (5.4) | 10.4 (9.9, 11.0) | 1.000 | 1.000 | 10.5 (10.0, 11.1) | 10.6 (9.9, 11.2) | 1.000 | 1.000 |
| n-6 fatty acids (%EI) | 4.9 (1.9) | 5.3 (5.0, 5.6) | 0.216 | 0.372 | 5.4 (5.1, 5.7) | 5.3 (5.0, 5.7) | 1.000 | 1.000 |
| Saturated fat (g) | 23.1 (14.3) | 25.0 (23.9, 26.1) | 1.000 | 1.000 | 24.8 (23.7, 25.9) | 24.3 (23.0, 25.5) | 0.429 | 1.000 |
| Saturated fat (%EI) | 12.8 ± 3.4 | 12.4 (11.9, 13.0) | 1.000 | 1.000 | 12.4 (11.8, 12.9) | 12.1 (11.4, 12.7) | 0.488 | 1.000 |
| Trans fat (g) | 1.0 (0.8) | 1.2 (1.1, 1.2) | 1.000 | 1.000 | 1.2 (1.1, 1.2) | 1.1 (1.0, 1.2) | 0.129 | 0.628 |
| Trans fat (%EI)^1^ | 0.5 (0.3) | 0.5 (0.5, 0.6) | 1.000 | 1.000 | 0.5 (0.5, 0.6) | 0.5 (0.5, 0.6) | 0.133 | 0.628 |
| Carbohydrate (g) | 207.1 (87.8) | 220.2 (214.5, 225.9) | 0.207 | 0.372 | 218.1 (212.4, 223.8) | 221.4 (214.7, 228.1) | 0.329 | 1.000 |
| Carbohydrate (%EI) | 47.8 ± 7.1 | 48.2 (47.0, 49.5) | 0.090 | 0.174 | 47.7 (46.4, 48.9) | 48.5 (47.0, 49.9) | 0.224 | 0.772 |
| NMES (g)^1^ | 45.6 (44.3) | 43.6 (39.2, 48.4) | 0.012^2^ | 0.034^3^ | 41.0 (36.9, 45.5) | 41.1 (36.3, 46.5) | 1.000 | 1.000 |
| NMES (%EI)^1^ | 10.7 (7.9) | 10.0 (9.1, 11.1) | 0.002^2^ | 0.010^3^ | 9.4 (8.5, 10.4) | 9.4 (8.3, 10.6) | 1.000 | 1.000 |
| Alcohol (g) | 3.0 (18.0) | 10.9 (7.4, 14.3) | 0.236 | 0.385 | 9.7 (6.2, 13.1) | 9.1 (5.0, 13.2) | 1.000 | 1.000 |
| Alcohol (%EI) | 1.2 (7.0) | 3.4 (2.3, 4.6) | 0.921 | 1.000 | 3.2 (2.1, 4.3) | 3.0 (1.7, 4.3) | 1.000 | 1.000 |
| NSP (g)^1^ | 13.0 (6.7) | 12.4 (11.8, 13.0) | <0.001^2^ | 0.003^3^ | 12.9 (12.2, 13.6) | 12.9 (12.2, 13.7) | 1.000 | 1.000 |
| Carbohydrate:NSP^1^ | 16.0 (6.5) | 17.0 (16.1, 18.0) | 0.001^2^ | 0.006^3^ | 16.3 (15.5, 17.3) | 16.7 (15.6, 17.8) | 0.902 | 1.000 |
| Folate(μg)^1^ | 226.5 (123.1) | 225.0 (212.3, 238.7) | 0.006 | 0.021^3^ | 233.5 (220.1, 247.4) | 243.7 (227.5, 260.9) | 0.122 | 0.628 |
| Vitamin B_12_ (μg)^1^ | 4.4 (3.0) | 4.3 (3.9, 4.7) | 0.017^2^ | 0.044^3^ | 4.5 (4.1, 4.9) | 4.4 (4.0, 4.9) | 1.000 | 1.000 |
| Vitamin C (mg)^1^ | 67.3 (62.4) | 61.6 (60.0, 69.1) | <0.001^2^ | 0.002^3^ | 67.4 (60.2, 75.6) | 69.3 (60.6, 79.2) | 1.000 | 1.000 |
| Potassium (mg) | 2686.4 (1114.6) | 2658.9 (2558.8, 2759.1) | 0.346 | 0.511 | 2690.7 (2590.4, 2791.0) | 2688.5 (2571.0, 2806.0) | 1.000 | 1.000 |
| Calcium (mg)^1^ | 757.8 (382.1) | 673.8 (641.6, 707.7) | 0.280 | 0.434 | 662.5 (630.8, 695.9) | 676.5 (638.4, 716.20) | 0.720 | 1.000 |
| Iron (mg) | 9.9 (4.6) | 10.4 (9.9, 10.8) | <0.001^2^ | <0.001^3^ | 10.7 (10.3, 11.2) | 10.8 (10.3, 11.3) | 1.000 | 1.000 |
| Fruits & Vegetables (g)^1^ | 247.7 (220.7) | 236.2 (209.8, 266.2) | <0.001^2^ | 0.002^3^ | 260.1 (231.1, 293.1) | 233.9 (203.6, 268.9) | 0.039^2^ | 0.405 |

Abbreviations: NMES (non-milk extrinsic sugars), %EI (% energy intake), NSP (non-starch polysaccharides)

ANCOVA was used adjusted for age, sex, body mass index (BMI), total energy intake (except for when assessing energy intake as the outcome), ethnicity, economic status, smoking status, alcohol intake (except when assessing alcohol as an outcome), number of children below 4 years, and long-standing illness.

^1^Residual data are non-normally distributed. The values presented from these variables are geometric (back log transformed) means with 95% CI.

^2^Two-sided *P*<0.05 shows a significant difference.

^3^Significant difference using multiple comparison-adjusted FDR p values using the Benjamini–Hochberg False Discovery Rate.

Supplementary Table 3. Food group consumers according to sleep duration in UK adults (19-64 y) participating in the NDNS 2008–2019 (n = 5,015).

|  | **Sleep duration group (N (%))** | | | **P value** | **FDR value** |
| --- | --- | --- | --- | --- | --- |
|  | **Short sleep, <7 h (n = 1,279)** | **Adequate sleep,** $\boldsymbol{\geq}$**7 - <9 h (n = 2,299)** | **Long sleep,** $\boldsymbol{\geq}$**9h (n = 272)** |  |  |
| Bacon and ham | 998 (58.3%) | 1734 (58.8%) | 184 (51.5%) | 0.030^1^ | 0.078 |
| Beef, veal and dishes | 802 (46.9%) | 1469 (49.8%) | 163 (45.7%) | 0.078 | 0.134 |
| Beer, lager, cider and perry | 508 (29.7%) | 865 (29.4%) | 79 (22.1%) | 0.013^1^ | 0.043^2^ |
| Biscuits | 1074 (62.8%) | 1855 (62.9%) | 203 (56.9%) | 0.077 | 0.134 |
| Brown granary and wheatgerm bread | 570 (33.3%) | 1055 (35.8%) | 100 (28.0%) | 0.007^1^ | 0.026^2^ |
| Buns, cakes, pastries and fruit pies | 807 (47.2%) | 1500 (50.9%) | 160 (44.8%) | 0.011^1^ | 0.039^2^ |
| Burgers and kebabs | 244 (14.3%) | 379 (12.9%) | 59 (16.5%) | 0.100 | 0.162 |
| Butter | 597 (34.9%) | 1078 (36.6%) | 122 (34.2%) | 0.406 | 0.530 |
| Cheese | 1005 (58.7%) | 1863 (63.2%) | 196 (54.9%) | <0.001^1^ | 0.004^2^ |
| Chicken and turkey dishes | 1138 (66.5%) | 2020 (68.5%) | 232 (65.0%) | 0.198 | 0.297 |
| Chips, fried roast potatoes and potato products | 1097 (64.1%) | 1844 (62.6%) | 228 (63.9%) | 0.554 | 0.665 |
| Chocolate confectionery | 733 (42.8%) | 1368 (46.4%) | 140 (39.2%) | 0.006^1^ | 0.024^2^ |
| Coated chicken | 267 (15.6%) | 474 (16.1%) | 60 (16.8%) | 0.825 | 0.853 |
| Crisps and savoury snacks | 808 (47.2%) | 1481 (50.3%) | 157 (44.0%) | 0.023^1^ | 0.066 |
| Dry weight beverages | 312 (18.2%) | 485 (16.5%) | 49 (13.7%) | 0.076 | 0.134 |
| Eggs and egg dishes | 840 (49.1%) | 1588 (53.9%) | 195 (54.6%) | 0.005^1^ | 0.021^2^ |
| Fruit | 1263 (73.8%) | 2283 (77.5%) | 234 (65.5%) | <0.001^1^ | <0.001^2^ |
| Fruit juice | 531 (31.0%) | 1079 (36.6%) | 119 (33.3%) | 0.001^1^ | 0.006^2^ |
| High fibre breakfast cereals | 774 (45.2%) | 1328 (45.1%) | 127 (35.6%) | 0.002^1^ | 0.011^2^ |
| Ice cream | 293 (17.1%) | 524 (17.8%) | 54 (15.1%) | 0.434 | 0.554 |
| Lamb and dishes | 215 (12.6%) | 408 (13.8%) | 34 (9.5%) | 0.053 | 0.114 |
| Liver dishes | 68 (4.0%) | 137 (4.6%) | 15 (4.2%) | 0.547 | 0.665 |
| Low fat spread polyunsaturated | 174 (10.2%) | 318 (10.8%) | 42 (11.8%) | 0.624 | 0.720 |
| Low fat spread not polyunsaturated | 88 (5.1%) | 121 (4.1%) | 17 (4.8%) | 0.251 | 0.359 |
| Meat pies and pastries | 379 (22.2%) | 663 (22.5%) | 85 (23.8%) | 0.791 | 0.837 |
| Nuts and seeds | 334 (19.5%) | 682 (23.1%) | 63 (17.6%) | 0.003^1^ | 0.015^2^ |
| Oily fish | 362 (21.2%) | 675 (22.9%) | 55 (15.4%) | 0.004^1^ | 0.018^2^ |
| One percent milk | 47 (2.7%) | 52 (1.8%) | 7 (2.0%) | 0.078 | 0.134 |
| Other bread | 170 (9.9%) | 289 (9.8%) | 31 (8.7%) | 0.765 | 0.835 |
| Other breakfast cereals | 444 (25.9%) | 874 (29.7%) | 96 (26.9%) | 0.022^1^ | 0.066 |
| Other margarine fats and oils | 181 (10.6%) | 373 (12.7%) | 32 (9.0%) | 0.026^1^ | 0.071 |
| Other meat and meat products | 224 (13.1%) | 426 (14.5%) | 37 (10.4%) | 0.07 | 0.134 |
| Other potatoes and potato salads dishes | 1082 (63.2%) | 1911 (64.8%) | 211 (59.1%) | 0.081 | 0.135 |
| Other milk and cream | 358 (20.9%) | 628 (21.3%) | 75 (21.0%) | 0.95 | 0.950 |
| Other white fish, shellfish and fish dishes | 518 (30.3%) | 1040 (35.3%) | 98 (27.5%) | <0.001^1^ | 0.002^2^ |
| Pasta, rice and other cereals | 1283 (75.0%) | 2322 (78.8%) | 259 (72.5%) | 0.001^1^ | 0.006^2^ |
| Pork and dishes | 335 (19.6%) | 580 (19.7%) | 56 (15.7%) | 0.189 | 0.291 |
| Puddings | 359 (21.0%) | 598 (20.3%) | 63 (17.6%) | 0.361 | 0.481 |
| PUFA margarine oils | 44 (2.6%) | 120 (4.1%) | 15 (4.2%) | 0.023^1^ | 0.066 |
| Reduced fat spread not polyunsaturated | 690 (40.3%) | 1268 (43%) | 134 (37.5%) | 0.05 | 0.114 |
| Reduced fat spread polyunsaturated | 208 (12.2%) | 378 (12.8%) | 40 (11.2%) | 0.601 | 0.707 |
| Salad and other raw vegetables | 1187 (69.4%) | 2224 (75.5%) | 219 (61.3%) | <0.001^1^ | <0.001^2^ |
| Sausages | 565 (33.0%) | 953 (32.3%) | 116 (32.5%) | 0.891 | 0.906 |
| Savoury sauces, pickles, gravies, and condiments | 1368 (80.0%) | 2486 (84.4%) | 268 (75.1%) | <0.001^1^ | <0.001^2^ |
| Semi skimmed milk | 1201 (70.2%) | 2160 (73.3%) | 232 (65.0%) | 0.001^1^ | 0.006^2^ |
| Skimmed milk | 258 (15.1%) | 429 (14.6%) | 45 (12.6%) | 0.482 | 0.603 |
| Soft drinks low calorie | 668 (39.0%) | 1216 (41.3%) | 126 (35.3%) | 0.053 | 0.114 |
| Soft drinks not low calorie | 774 (45.2%) | 1388 (47.1%) | 185 (51.8%) | 0.067 | 0.134 |
| Soup homemade and retail | 485 (28.3%) | 896 (30.4%) | 112 (31.4%) | 0.264 | 0.368 |
| Spirits and liqueurs | 211 (12.3%) | 385 (13.1%) | 30 (8.4%) | 0.041^1^ | 0.103 |
| Sugar confectionery | 248 (14.5%) | 448 (15.2%) | 57 (16.0%) | 0.706 | 0.784 |
| Sugars, preserves and sweet spreads | 1064 (62.2%) | 1892 (64.2%) | 236 (66.1%) | 0.234 | 0.342 |
| Tea, coffee and water | 1689 (98.7%) | 2899 (98.4%) | 346 (96.9%) | 0.05 | 0.114 |
| Vegetables not raw | 1490 (87.1%) | 2652 (90%) | 299 (83.8%) | <0.001^1^ | 0.002^2^ |
| White bread | 1315 (76.9%) | 2289 (77.7%) | 278 (77.9%) | 0.795 | 0.837 |
| White fish coated or fried | 322 (18.8%) | 583 (19.8%) | 72 (20.2%) | 0.685 | 0.775 |
| Wholemeal bread | 560 (32.7%) | 1022 (34.7%) | 115 (32.2%) | 0.318 | 0.434 |
| Whole milk | 363 (21.2%) | 570 (19.3%) | 81 (22.7%) | 0.149 | 0.235 |
| Wine | 479 (28.0%) | 945 (32.1%) | 83 (23.2%) | <0.001^1^ | 0.002^2^ |
| Yogurt, fromage, frais and dairy desserts | 622 (36.4%) | 1153 (39.1%) | 123 (34.5%) | 0.067 | 0.134 |

Data are presented as N (%).

Categorical data were tested by Chi-square test. *P*<0.05 indicates a significant association between sleep duration and the food groups.

^1^Two-sided *P*<0.05 shows a significant difference.

^2^Significant difference using multiple comparison-adjusted FDR p values using the Benjamini–Hochberg False Discovery Rate.

Supplementary Table 4. Biomarkers of nutrient intakes (estimated marginal mean (95% CI)) according to social jetlag status in a representative UK adult population (19-64 y) based on NDNS 2008–2019 (n=5,015).

| **Dietary intake** | **No social jetlag** | **Social jetlag** | ***P* value** | **FDR value** |
| --- | --- | --- | --- | --- |
| Vitamin B_12_ (pmol/l)^2^ | 261.4 (238.7, 286.3) | 254.9 (231.6, 280.6) | 0.203 | 0.508 |
| Vitamin C (μmol/l) | 62.5 (49.4, 75.6) | 63.8 (50.1, 77.6) | 0.633 | 0.961 |
| Haemoglobin (g/dl) | 13.8 (13.5, 14.1) | 13.9 (13.6, 14.1) | 0.101 | 0.281 |
| Serum Ferritin (μg/l)^2^ | 64.2 (45.7, 90.2) | 62.6 (44.3, 88.5) | 0.618 | 0.961 |
| Urinary Na^+1^ excretion (mmol/24hrs) | 115.5 (99.2, 131.7) | 117.9 (99.5, 136.3) | 0.602 | 0.961 |
| Urinary K^+^ excretion (mmol/24hrs)^2^ | 60.3 (54.1, 67.4) | 59.6 (52.6, 67.4) | 0.661 | 0.961 |
| Urinary N^3-^ excretion (g/24hrs)^2^ | 10.8 (9.9, 11.8) | 10.6 (9.8, 12.0) | 0.845 | 0.961 |

Vitamin B_12_ (pmol/l): without social jetlag n=1873; with social jetlag n=310; vitamin C (μmol/l): without social jetlag n=700; with social jetlag n=110; haemogloblin: without social jetlag n=1861; with social jetlag n=316; serum ferritin: without social jetlag n=1880; with social jetlag n=309; urinary K^+^, N^3^ and Na^+1^: without social jetlag n=1018; with social jetlag n=147.

ANCOVA was used adjusted for age, sex, body mass index (BMI), ethnicity, economic status, smoking status, alcohol intake, number of children below 4 years, and long standing illness.

^2^Residual data are non-normally distributed. The values presented from these variables are geometric (back log transformed) means with 95% CI.

Supplementary Table 5. Food group consumers according to social jetlag status in a representative UK adult population sample (19-64 y) from the NDNS 2008–2019 (n = 5,015).

| **Food groups** | **N (%)** | | | ***P* value** | **FDR value** |
| --- | --- | --- | --- | --- | --- |
|  | **No social jetlag (n_max_^1^ = 4,307)** | **Social jetlag (n_max_^1^ = 708)** |  | |  |
| Bacon and ham | 2485 (57.7%) | 431 (60.9%) | 0.112 | | 0.339 |
| Beef, veal and dishes | 2083 (48.4%) | 351 (49.6%) | 0.549 | | 0.732 |
| Beer lager cider perry | 1238 (28.7%) | 214 (30.2%) | 0.420 | | 0.681 |
| Biscuits | 2694 (62.5%) | 438 (61.9%) | 0.727 | | 0.846 |
| Brown granary and wheat germ bread | 1483 (34.4%) | 242 (34.2%) | 0.896 | | 0.977 |
| Buns, cakes, pastries, fruit pies | 2142 (49.7%) | 325 (45.9%) | 0.059 | | 0.229 |
| Burgers and kebabs | 586 (13.6%) | 96 (13.6%) | 0.973 | | 0.989 |
| Butter | 1552 (36%) | 245 (34.6%) | 0.462 | | 0.688 |
| Cheese | 2639 (61.3%) | 425 (60.0%) | 0.529 | | 0.732 |
| Chicken and turkey dishes | 2906 (67.5%) | 484 (68.4%) | 0.639 | | 0.833 |
| Chips, fried roast potatoes and potato products | 2699 (62.7%) | 470 (66.4%) | 0.057 | | 0.229 |
| Chocolate confectionery | 1876 (43.6%) | 365 (51.6%) | <0.001^1^ | | 0.003^2^ |
| Coated chicken | 671 (15.6%) | 130 (18.4%) | 0.061 | | 0.229 |
| Crisps and savoury snacks | 2053 (47.7%) | 393 (55.5%) | <0.001^1^ | | 0.003^2^ |
| Dry weight beverages | 723 (16.8%) | 123 (17.4%) | 0.699 | | 0.846 |
| Eggs and egg dishes | 2286 (53.1%) | 337 (47.6%) | 0.007^1^ | | 0.053 |
| Fruit | 3277 (76.1%) | 503 (71.0%) | 0.004^1^ | | 0.034^2^ |
| Fruit juice | 1489 (34.6%) | 240 (33.9%) | 0.727 | | 0.846 |
| High fibre breakfast cereals | 1925 (44.7%) | 304 (42.9%) | 0.383 | | 0.657 |
| Ice cream | 755 (17.5%) | 116 (16.4%) | 0.456 | | 0.688 |
| Lamb and dishes | 573 (13.3%) | 84 (11.9%) | 0.293 | | 0.533 |
| Liver dishes | 187 (4.3%) | 33 (4.7%) | 0.701 | | 0.846 |
| Low fat spread polyunsaturated | 469 (10.9%) | 65 (9.2%) | 0.172 | | 0.408 |
| Low fat spread not polyunsaturated | 187 (4.3%) | 39 (5.5%) | 0.165 | | 0.408 |
| Meat pies and pastries | 954 (22.1%) | 173 (24.4%) | 0.177 | | 0.408 |
| Nuts and seeds | 926 (21.5%) | 153 (21.6%) | 0.947 | | 0.989 |
| Oily fish | 967 (22.5%) | 125 (17.7%) | 0.004^1^ | | 0.034^2^ |
| One percent milk | 91 (2.1%) | 15 (2.1%) | 0.992 | | 0.992 |
| Other bread | 435 (10.1%) | 55 (7.8%) | 0.053 | | 0.229 |
| Other breakfast cereals | 1211 (28.1%) | 203 (28.7%) | 0.761 | | 0.846 |
| Other margarine fats and oils | 519 (12.1%) | 67 (9.5%) | 0.047^1^ | | 0.229 |
| Other meat and meat products | 558 (13%) | 129 (18.2%) | <0.001^1^ | | 0.003^2^ |
| Other potatoes potato salads dishes | 2796 (64.9%) | 408 (57.6%) | <0.001^1^ | | 0.003^2^ |
| Other milk and cream | 922 (21.4%) | 139 (19.6%) | 0.284 | | 0.533 |
| Other white fish shellfish fish dishes | 1432 (33.2%) | 224 (31.6%) | 0.399 | | 0.665 |
| Pasta, rice and other cereals | 3311 (76.9%) | 553 (78.1%) | 0.470 | | 0.688 |
| Pork and dishes | 840 (19.5%) | 131 (18.5%) | 0.532 | | 0.732 |
| Puddings | 882 (20.5%) | 138 (19.5%) | 0.546 | | 0.732 |
| PUFA margarine oils | 159 (3.7%) | 20 (2.8%) | 0.249 | | 0.533 |
| Reduced fat spread not polyunsaturated | 1806 (41.9%) | 286 (40.4%) | 0.442 | | 0.688 |
| Reduced fat spread polyunsaturated | 538 (12.5%) | 88 (12.4%) | 0.963 | | 0.989 |
| Salad and other raw vegetables | 3137 (72.8%) | 493 (69.6%) | 0.077 | | 0.257 |
| Sausages | 1386 (32.2%) | 248 (35.0%) | 0.134 | | 0.365 |
| Savoury sauces pickles gravies condiments | 3531 (82.0%) | 591 (83.5%) | 0.336 | | 0.593 |
| Semi skimmed milk | 3106 (72.1%) | 487 (68.8%) | 0.068 | | 0.240 |
| Skimmed milk | 628 (14.6%) | 104 (14.7%) | 0.940 | | 0.989 |
| Soft drinks low calorie | 1708 (39.7%) | 302 (42.7%) | 0.131 | | 0.365 |
| Soft drinks not low calorie | 1985 (46.1%) | 362 (51.1%) | 0.013^1^ | | 0.087 |
| Soup homemade and retail | 1286 (29.9%) | 207 (29.2%) | 0.738 | | 0.846 |
| Spirits and liqueurs | 529 (12.3%) | 97 (13.7%) | 0.290 | | 0.533 |
| Sugar confectionery | 644 (15.0%) | 109 (15.4%) | 0.760 | | 0.846 |
| Sugars preserves and sweet spreads | 2754 (63.9%) | 438 (61.9%) | 0.287 | | 0.533 |
| Tea, coffee and water | 4248 (98.6%) | 686 (96.9%) | 0.001^1^ | | 0.012^2^ |
| Vegetables not raw | 3831 (88.9%) | 610 (86.2%) | 0.031^1^ | | 0.186 |
| White bread | 3323 (77.2%) | 559 (79.0%) | 0.288 | | 0.533 |
| Whitefish coated or fried | 820 (19.0%) | 157 (22.2%) | 0.051 | | 0.229 |
| Wholemeal bread | 1472 (34.2%) | 225 (31.8%) | 0.212 | | 0.471 |
| Whole milk | 867 (20.1%) | 147 (20.8%) | 0.698 | | 0.846 |
| Wine | 1310 (30.4%) | 197 (27.8%) | 0.163 | | 0.408 |
| Yogurt, fromage frais and dairy desserts | 1649 (38.3%) | 249 (35.2%) | 0.113 | | 0.339 |

Data are presented as N (%).

Categorical data were tested by Chi-square test. *P*<0.05 indicates a significant association between social jetlag and the food groups.

^1^Two-sided *P*<0.05 shows a significant difference.

^2^Significant difference using multiple comparison-adjusted FDR p values using the Benjamini–Hochberg False Discovery Rate.
